# Factors Affecting Well-Being and Quality of Life From the Perspective of People With Moderate-To-Severe Dementia: A Qualitative Evidence Synthesis

**DOI:** 10.1177/14713012251410993

**Published:** 2025-12-29

**Authors:** Claire Pentecost, Anna Carr, Catherine M. Alexander, Linda Clare

**Affiliations:** 1 REACH: The Centre for Research in Ageing and Cognitive Health, College of Medicine and Health, 3286 University of Exeter, Exeter, UK; 2 NIHR PenARC (National Institute for Health and Care Research, Applied Research Collaboration South-West Peninsula), UK

**Keywords:** psychological health, moderate-to-severe dementia, life satisfaction, systematic review, residential care, care home

## Abstract

**Objectives:** Dementia care should meet the mental, physical, and daily care needs of people who are less able to take care of themselves. Research has been undertaken to understand the subjective perspectives of well-being and quality of life in people with mild-to-moderate dementia, but there is less clarity about the desires and influences on well-being of people with moderate-to -severe dementia. We aimed to understand what is important for well-being or quality of life from the perspective of people with moderate-to-severe dementia. **Methods:** We searched published literature and conducted a systematic review and qualitative thematic synthesis. We included qualitative studies that presented views on and experiences of well-being or quality of life from the perspectives of people with moderate-to-severe dementia. **Findings:** We included five studies. All were conducted in residential care homes, and data collection was carried out using face-to-face semi-structured conversational interviews with people with moderate-to-severe dementia. Three themes were identified: how people felt about their lives in the care home, how they felt about the care home itself, and how they felt about themselves. The overarching aspect of these three themes was the ability and opportunity to experience positive associations with past and present personal identity. **Conclusions:** People with moderate-to-severe dementia can communicate their preferences and wishes and need support to spend their time in ways that support their quality of life. Opportunities for positive reminiscence, daily activities that can enhance moments of pleasure, and positive reinforcement of identity can help maintain well-being and quality of life. Care home staff and family members may benefit from education and training in these aspects of care.

## Introduction

Over 55 million people are living with dementia worldwide, and this figure is rapidly rising (World Health Organization, 2021). Acknowledging the urgency of addressing the needs of more people living with dementia worldwide, the World Health Organization has called for dementia care that aims for people with dementia and their carers to “live well and receive the care and support they need to fulfil their potential with dignity, respect, autonomy, and equality” (World Health Organization, 2017, p. 6). The term ‘live well’ acknowledges the need to go beyond thinking only of illness and shift towards a more holistic and whole-person approach to inform care and support for people living with dementia. Understanding what it means to live well with dementia at any stage of the disease is essential to inform public understanding and enable carers, service providers, and policymakers to make the best possible decisions about care and support for people with dementia.

In a previous study the IDEAL (Improving the experience of Dementia and Enhancing Active Life) programme (Clare et al., 2014) focused on living well for people with mild-to-moderate dementia in the community, and their unpaid carers, and reciprocal influences of the well-being of each on the other. This was a British cohort study with a sample of 1537 people with dementia and 1266 of their unpaid carers recruited at baseline. Living well was explored through measures of quality of life, well-being, and satisfaction with life collected at three timepoints at 12-month intervals, face-to-face in participants’ homes. This yielded valuable insights about what contributes to a sense of living well.

Cross-sectional analyses of the IDEAL cohort data (Clare et al., 2019) showed that for people with mild-to-moderate dementia, a more positive social situation, greater social resources, better physical health, better ability to manage everyday life with dementia, and better psychological health were all associated with higher living well scores and vice versa. When looking at the relative influences of these factors, we found that psychological health was most closely related to perceptions of living well. This suggested that social situation, social resources, physical health, and ability to manage everyday life all influenced psychological health, which in turn was strongly linked to perceptions of living well. For people with mild-to-moderate dementia in the community, poor psychological health, depression, loneliness, and a sense of being in poorer physical health are all signs that there is a risk of declining capability to live well (Clare et al., 2022).

We aimed to extend our work to consider living well for people with moderate-to-severe dementia. We wanted to see to what extent the model arising from these findings was relevant to people with moderate-to-severe dementia, and whether other factors came into play as dementia severity progressed. Research since the 1900s has confirmed that people with moderate-to-severe dementia have capacity for emotional expression (Kitwood & Bredin, 1992; Magai et al., 1996) and can reliably report their own mood (Kolanowski et al., 2002) and quality of life consistently (Logsdon et al., 1999; Thorgrimsen et al., 2003). It is therefore important to attempt to gain access to the insights and experiences of people with more advanced dementia so we can understand how to assist them to fulfil their potential with dignity. We wanted to explore the subjective perspectives of living well and to seek insights about what matters to the individual.

However, relatively few studies have attempted to elicit the subjective experiences of people whose dementia has progressed beyond the mild-to-moderate stage. A review of qualitative studies that aimed to identify factors that influence quality of life from the perspective of people with dementia of all severity levels (O’Rourke et al., 2015) included 11 studies published between 1975 and 2012. The review identified five constructs associated with well-being: connectedness, relationships, agency in life today, wellness, and a sense of place, all of which were consistent with the IDEAL model. Four of the 11 studies included participants with moderate-to-severe dementia, although only one (Clare et al., 2008) presented verbatim quotes from participants, so it remains unclear which of these constructs apply specifically to people with moderate-to-severe dementia. The constructs of agency in life today and a sense of place could be especially relevant for people in the later stages of dementia who have moved from their home to live in residential care.

This review aims to extend understanding of the subjective experience of well-being and quality of life to those with moderate-to-severe dementia. The review question is: what is important for well-being or quality of life from the perspective of the person with moderate-to-severe dementia?

## Methods

This study is a systematic review and qualitative thematic synthesis. The ENTREQ checklist (Tong et al., 2007) has been used as a reporting framework for this study. The completed checklist can be found in Supplemental file 1. The study is registered in PROSPERO CRD42023427995.

### Search Strategy

The following databases were searched on 21^st^ March 2025 through the OVID platform: Embase, Ovid Medline, APA PsycInfo, and Social Policy and Practice. Search terms were constructed to identify all studies that recruited people with any kind of dementia, from community or residential care settings, and that explored quality of life, well-being, or satisfaction with life using qualitative methods. English language filters were applied, and there were no date restrictions. Qualitative filter terms were derived from advice found on the InterTASC Information Specialists’ Sub-Group Search Filter Resource for qualitative research https://sites.google.com/a/york.ac.uk/issg-search-filters-resource/home/qualitative-research. The search terms can be found in Supplemental file 2.

### Inclusion Criteria

Studies were included if they focused on people with moderate-to-severe dementia or indicated that at least two-thirds of participants with dementia were in the moderate-to-severe stages, elicited subjective views about and experiences of well-being or quality of life using qualitative methods, and presented quotes from people with moderate-to-severe dementia. We included studies that recruited participants living in the community or residential long-term care settings, but excluded studies set in acute hospitals, as severe ill-health and the hospital setting are likely to have distinct implications for quality of life.

### Screening Procedure

We prepared a proforma setting out clear definitions to support the implementation of the inclusion and exclusion criteria and tested it with a random selection of the first one hundred articles identified in the search by CP and AC. It was then amended and agreed with the wider team to maximise screening accuracy.

After duplicates were removed, two researchers (CP and AC) independently screened titles and abstracts. Disagreements were identified, discussed, and resolved. Any cases of disagreement were referred to a third team member (LC) for resolution. If an article did not specify the severity of dementia but met all other inclusion criteria, the paper was put forward for full-text screening to allow us to identify any information that could ascertain the likely severity of the participants and reach an appropriate decision. Full-text versions of all potentially eligible studies were retrieved. Each study was independently assessed by two researchers (CP and AC), and disagreements were resolved by a third team member (LC). Studies not meeting inclusion criteria were filed, and the reasons for exclusion were noted. The reference sections of studies included at full-text review and relevant reviews were backward searched for other studies that might be suitable for inclusion (CP, CA). Abstracts and, if appropriate, full-text versions of these were evaluated as above.

### Critical Appraisal

The included studies were evaluated independently for relevance and methodological quality using the CASP Qualitative Studies Checklist (Critical Appraisal Skills Programme, 2023) by two researchers (CP, CA). Each paper was given a rating of yes, no, or unclear (data difficult to interpret or not present), and the ‘yes’ answers were added together to give a score out of a possible ten. The evaluation was used to identify studies that may provide unreliable findings or conclusions. Two reviewers compared scores and came to a consensus on the scoring. The quality assessment findings are reported in the results section.

### Data Extraction

Data extraction for qualitative synthesis was guided by the aim of our research and our research question (Popay et al., 2006). The findings and discussion sections of studies were copied and pasted directly into NVivo14 version 14.23.1 (Lumivero, 2023) for analysis.

### Analysis

Our approach to synthesising the included studies was informed by thematic syntheses of qualitative data (Thomas & Harden, 2008). Thematic synthesis is an interpretative approach to reviewing based on the methods of thematic analysis used in primary research. The thematic synthesis aimed to go beyond thematic analysis methods to enable new insights, interpretations, and theory to be developed, and to facilitate ‘translating’ - taking concepts from one study and recognising the same concepts in another study.

Data extraction, analysis, synthesis, and writing were iterative phases, involving movement backward and forward between them. Researchers gained familiarity by reading all papers thoroughly before moving to coding.

To ensure the analysis was based on the voices of people with dementia, initial coding focused on coding the quotes provided by authors from interviews with people with moderate-to-severe dementia inductively. Researchers (CP and AC) coded text line by line within NVivo14 (Lumivero, 2023) independently. Similarities and differences between codes were discussed and codes were developed into descriptive themes and viewed within a matrix using NVivo14 (Lumivero, 2023). Researchers (CP, LC) then developed analytical themes to attempt to go beyond the findings presented by authors in the original studies and to answer the review question. This was done by testing inferences in the original data, reviewing, discussing, and then agreeing on adjustments to themes. This process was repeated until the analytical themes sufficiently explained the descriptive coding generated by CP and AC.

The themes and interpretations reported by authors in the included studies were then organised against our findings to identify similarities and differences. Finally, overarching themes were developed to encompass the analytic themes in areas of well-being and quality of life most commonly raised by people with moderate-to-severe dementia.

## Results

Of 8045 records identified from databases and citation searching, five journal articles were included in the review. See the study selection process flow diagram in Figure 1.

**Figure 1.**
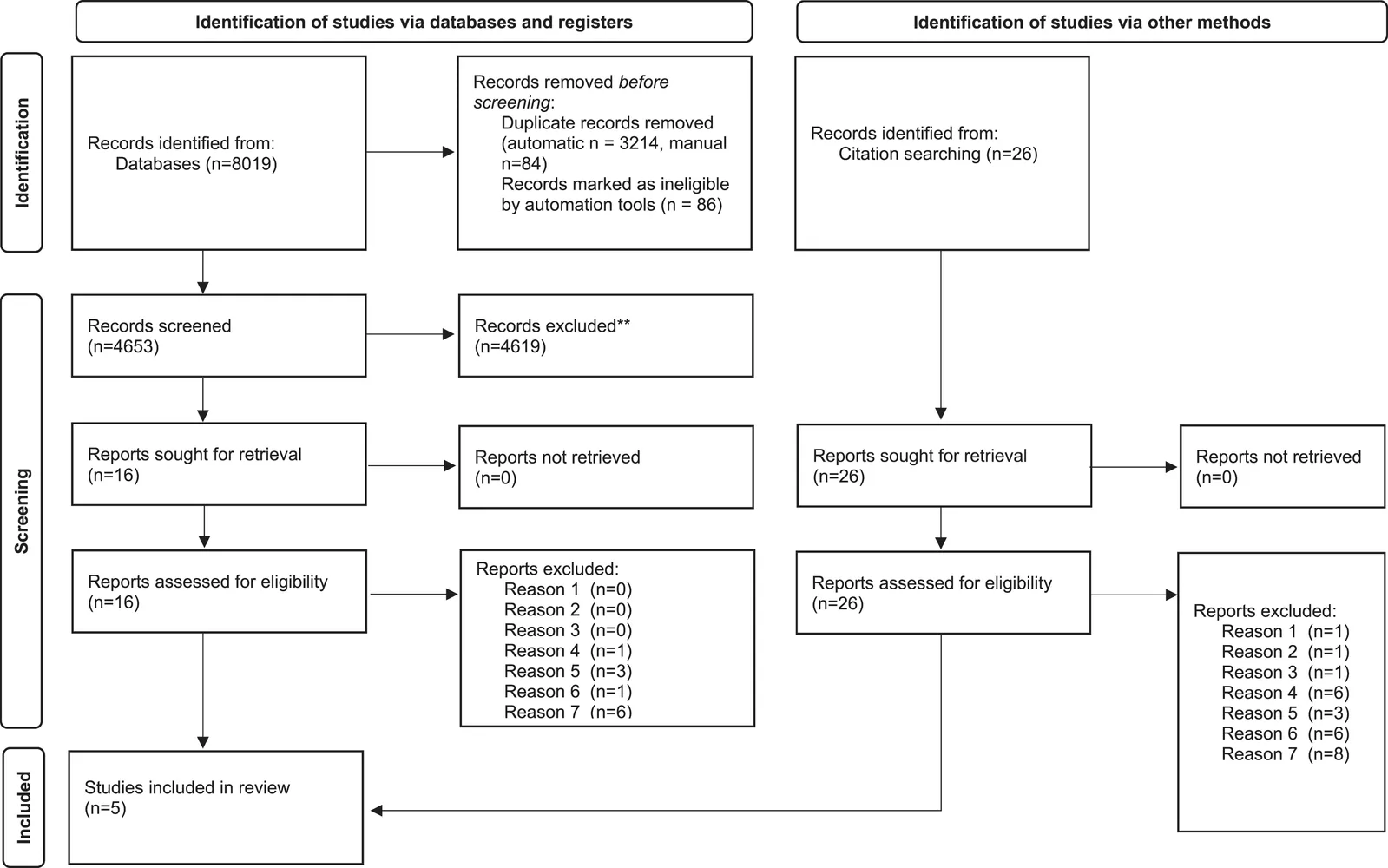
Flow diagram indicating the study selection process Exclusion criteria: 1 Not English language, 2 Not dementia, 3 Mild dementia or mild cognitive impairment only, 4 Severity not reported, 5 No qualitative data from people with moderate-to-severe dementia, 6 Not about quality of life, 7 Not majority moderate-to-severe dementia

All five studies involved recruiting and interviewing people with dementia living in nursing homes (Cahill & Diaz-Ponce, 2011, Mjørud et al., 2017; Nygaard et al., 2020; Verloo et al., 2018) or residential care homes (Clare et al., 2008). There was one study each from England and Wales, Ireland, and Switzerland, and two from Norway.

Dementia severity was assessed in four of the included studies using either the Mini-Mental State Evaluation (Arevalo-Rodriguez et al., 2015) or the Clinical Dementia Rating (Berg, 1984). One study did not assess dementia severity (Nygaard et al., 2020), but we included the paper based on the description of the residential care setting and participants as per the inclusion criteria. Across the studies, 194 people with dementia were recruited from 20 care homes, and 180 people had moderate-to-severe dementia. The mean age of participants with moderate-to-severe dementia across all studies was 85.2 years. Three studies were exploratory qualitative studies and two (Clare et al., 2008; Verloo et al., 2018) were mixed methods studies with a qualitative component. Data were collected between 1999 and 2018. A summary of extracted descriptive information from the included studies can be found in Supplemental file 3.

### Critical Appraisal

The overall relevance and methodological rigor of the studies were very good. A summary of the critical appraisal can be found in Table 1, and the detailed assessment is available in Supplemental file 4.

**Table 1.** Critical Appraisal Results

Lead author, year	1. Was there a clear statement of the aims of the research?	2. Is a qualitative methodology appropriate?	3. Was the research design appropriate to address the aims of the research?	4. Was the recruitment strategy appropriate for the aims of the research?	5. Was the data collected in a way that addressed the research issue?	6. Has the relationship between the researcher and the participants been adequately considered?	7. Have ethical issues been taken into consideration?	8. Was the data analysis sufficiently rigorous?	9. Is there a clear statement of findings?	10. How valuable is the research?	Score
Cahill and Diaz-Ponnce (2011)	Yes	Yes	Yes	Yes	Yes	Unclear	Yes	Yes	Yes	Yes	9
Clare et al. (2008)	Yes	Yes	Yes	Yes	Yes	Unclear	Unclear	Yes	Yes	Yes	8
Mjorud et al. (2017)	Yes	Yes	Yes	Yes	Yes	Unclear	Yes	Unclear	Yes	Yes	8
Nygaard et al. (2020)	Yes	Yes	Yes	Yes	Yes	Unclear	Yes	Yes	Yes	Yes	10
Verloo et al. (2018)	Yes	Yes	Yes	Yes	Yes	No	Yes	Unclear	Yes	Yes	8

Assessments were made using the CASP Qualitative Studies Checklist (Critical Appraisal Skills Programme, 2023).

### Qualitative Synthesis Findings

All studies reported a range of positive and negative experiences relevant to well-being. The themes developed by the study authors are grouped according to our analytical themes in Table 2.

**Table 2.** Author Themes Categorised Under Analytical Themes

Analytical themes	Social connection and feeling connected		Meaningful engagement	Affect	Coping	Identity
Cahill and Diaz-Ponce (2011)	**Social contact **family, residents and staff	**Attachment **feeling at home/missing home and connectedness	**Pleasurable activities **(structured vs unstructured, momentary pleasures)	**Affect **(happiness, sadness, depression and loneliness)		
Clare et al. (2008)	**Nothing’s right now **isolation, loss, loneliness	**It drives me mad **annoyance with others	**It drives me mad **boredom	**Nothing’s right now **suffering uncertainty, worthlessness, fear **It drives me mad **frustration with oneself, anger, wanting more freedom	**I’m alright I’ll manage **acceptance, ways of coping, making the best of things	**I still am somebody** a sense of pride, affirming identity, managing well despite limitations
Mjørud et al. (2017)	**Everything is gone** Loss of family and home	**Things that make it better and things that make it worse **people **People - for better or worse **staff treatment, annoying, not being alone, family visits, residents not important	**Things that make it better and things that make it worse **activities are boring, enjoyment		**Being in the nursing home is okay, but you must take things as they are **contentedness, missing home, acceptance	**Everything is gone** Loss of past lives, loss of self **Things that make it better and things that make it worse **belongings, reminiscence
Nygaard et al. (2020)	**Myself and my relationship with fellow residents **friendship, little in common, trouble	**Creation of individualised living spaces** care and protection, security **Single rooms with personal décor that enhances a sense of connectedness** Feel like home, belongings, privacy **Transition from old home to new home **difficulty understanding, accepting, adapting	**Significant activities providing meaning **reflecting past and current interests, lack of activities, lack of freedom			**Myself and my relationship with fellow residents** single rooms with personal decor that enhances a sense of connectedness
Verloo et al. (2018)	**Human dignity and acceptance **social interactions, relationships, kind carers, lack of care		**Development and existence **moments to feel pleasure, interactions, activities, natural environment		**Recognition and safety **respect for private lives and physical privacy	**Functionality and health **maintaining physical and psychological health, maintaining skills

Themes from included studies are shown in **Bold**, and adjectives used by study authors to describe themes are in normal font.

We synthesised the analytical themes into three key areas of influence on well-being reflected in the accounts of people with moderate-to-severe dementia. An overview of the final themes and exemplar quotes can be found in Table 3. The themes are: *How they felt about their lives in the care home*, with sub-themes ‘I don’t like it but what can I do’, ‘Where I live is important to me’, and ‘Need for connection’; *How they felt about the care home itself*, with sub-themes, ‘People’, ‘Meaningful engagement’ and ‘Environment’ and *How they felt about themselves*, with subthemes ‘It’s not me’, and ‘I still can’.

**Table 3.** Final Themes and Exemplar Quotes

**How they felt about their lives in the care home**	
I don’t like it but what can I do	“Also, I can no longer cope with housekeeping and such things.” (P31) Nygaard
Where I live is important to me	“Personally what I want, is peace and quiet. (. . .) In general, it is quite good.” (female, 92) Cahill
Need for connection	“To get a hug when he leaves and a kiss when he leaves again… then the day is saved.” (Betty) Mjørud
**How they felt about the care home itself**	
People	“I’d rather do without knowing them [residents], you know, you know what I mean.” (GC01) Clare
Meaningful engagement	“Activities […] I enjoy going to them” (CIOA 2) “I need to go out to see the garden and the sheep.” (CIOA 11) Verloo
Environment	“My doll I have with me too. … I also arrange my flowers here. … We have some of our furniture here, and … we can’t have it better.” (P9) Nygaard
**How they felt about themselves**	
It’s not me	“I used to be er...a bloke that knew different things and all that, you know...I’ve lost it...Really lost it.” (MB05) Clare
I still can	“It is not home, but I go around dancing and enjoying myself. I do that because I must live my life…” (Mina) Mjørud

Example quotes from people with moderate or severe dementia are provided in the narrative below. The participant identifier used by authors is shown in brackets and the name of the lead author follows.

#### How People Feel About Their Lives in the Care Home

##### I Don’t Like It But What Can I Do?

Moving into a care home was seen as an unwelcome transition in people’s lives associated with a sense of loss; however, four of the five studies reported attempts to accept the situation. There was evidence of an understanding that they could no longer live in their own home and a pragmatic rationalisation of needing special care.

“And I don’t like it. But what can I do?” (GW02) Clare

“Yes, I would rather be at home, but I probably couldn’t.” (Mina) Mjørud

“[I] need to be in the hospital [home].” (Mary) Cahill.

Across three studies (Cahill, Clare, Mjørud), several people with dementia expressed gratitude and contentment for their lives in the care home, and the move may have been a positive change.

“It is good for me and I am happy. I just love being in here and I am so grateful. I don’t have anything to be worried about.” (female, 76) Cahill

Others were less happy and longed for their old lives and homes. There could be a lack of understanding of why they were in the home, and some feelings of being ‘put away’ (Nygaard).

‘‘I don’t know whether I’m stuck here for the rest of me life or what’s happening really.’’ (GR03) Clare

##### Where I Live Is Important To Me

A sense of home could mean many things to people, but the loss of home and the people associated with it, compared to being surrounded by people they were not emotionally attached to, could contribute to a feeling of disconnection.

‘‘I wouldn’t say it was as good as home, like. . . er . . . you haven’t got that same feeling or love or anything is. . . a . . . at a place like this. You’re just. . . one of a number, group, who are pretty well in a similar position.” (CB04) Clare

Whether people felt at home or not resonated across all studies. The studies reported some people expressed feeling at home in the care home and others wanted to leave or believed that staying in the care home was temporary.

“I hope I’ll be, get loose one of these days.” (AL06) Clare

“I think it is alright, but not all the time, no I don’t want that.” (Mina) Mjørud

Believing that the care home was not their permanent residence could explain their confusion about where they were, and perhaps ease discomfort at the thought that they were no longer at home, reflected in the quote from Nygaard.

“In a way, I feel slightly at home, but now, I’m so forgetful that I don’t remember if it’s Dad or me who was here. Who owns this room in a way?” (P15) Nygaard

##### Need for Connection

Across all studies, there was a firm acknowledgment that social connection was important for well-being, and this was presented in talk about positive social attachments and loneliness. Being in the company of other residents could be a source of connection or friendship.

“I have my door open, and I look straight at the patients. … If I were to just sit with the door shut, I don’t know what would have happened.” (P15) Nygaard

However, the people most often talked about were family members. Missing family was a source of sadness, loneliness, and longing, exacerbated by not remembering that people had visited and uncertainty about when they would see them again.

“They don’t bother visiting me.” (Female, 87) Cahill

“I miss my sister.” (Lisa) Mjørud

People with dementia were happiest around people they loved and recalled visits with enthusiasm.

“They come to visit me; we enjoy being together and chatting.” (12) Verloo

Two people quoted in the Cahill paper also expressed missing their wider social community.

“I miss home and my friends, the neighbours.” (female, 81) Cahill

“I’d like to be able to talk to people, where people gather in the community.” (male, 74) Cahill

One study reported evidence of distress linked to fear of being alone.

“I’m frightened, I’m frightened, please help me, I’m frightened of being alone.” (GW02) Clare

“Don’t lose me will you.” (WG05) Clare

#### How People Feel About the Care Home Itself

##### People

Residents rarely mentioned attachment to staff or residents. One man mentioned he was particularly fond of a staff member, but the author pointed out that this was the only person who had spoken to him that day (Cahill). People tended to speak about the staff as ‘kind’ and ‘nice’ and looking after them well, although they disliked staff who did not respect their privacy and thought the staff were absent too often.

“People who get involved in all sorts of things that aren’t their business.” (9) Verloo

“He runs off, you see.” (Peter) Mjørud

Examples of interactions indicated that staff were not attending to residents’ emotional needs.

“[I] got the feeling I am just a brat she has to take care of.” (Marta) Mjørud

“I don’t know anybody in here. I spend my day in the bed.” (female, 92) Cahill

The other residents could either be considered a source of connection or a source of annoyance. Two people explicitly mentioned enjoying the company of other residents.

“I get on very well, I sit in the piano room which is quieter and say hello to everybody.” (female, 84) Cahill

‘‘I’ve got a pal, she helps me out.’’ (MP01) Clare

In contrast, others only tolerated other residents, and sometimes there was deep frustration when other residents were disruptive or non-responsive. People with dementia did not feel they had full autonomy about moving into the home; most wanted to be in their own home, and experienced a lack of control because they lacked choice about who they were now mixing with.

“I get so annoyed when I sit and listen to their fuss in their conversations at the tables and stuff.” (P31) Nygaard

However, even if they had not formed friendships, a feeling of acceptance could be important.

“But they have been friendly, it’s been nice. And I think I’ve got in, at last.” (GC01) Clare

##### Meaningful Engagement

The possibility of engaging in meaningful activities whether done privately or in a group was important for well-being (Cahill, Clare, Nygaard, Verloo). Having interesting or enjoyable things to do helped to reduce boredom, provided opportunities to interact with people and allowed the person with dementia to re-connect with themselves.

The types of activities enjoyed by residents were numerous and could be organised group activities or activities the residents could choose to do alone in their own time. An account of enthusiastic participation in activities with other people in the home indicated a desire for contact with other people.

“We have thrown such big balls between us. [It is] very nice when you get your arms touched then. It is very nice. Also, we have had games such as Ludo.” (P7) Nygaard

Several of the homes provided creative activities that could lift mood.

“manual activities … building a table…” (5) Verloo

“Is painting an activity you often do?” “Often.” (16) Verloo

“Also, you should not be so careful if it is right what you sing. You forget, but you get in a little better mood, at least I do.” (P19) Nygaard

People liked to have options to do things during their private quiet time, doing normal things such as “solving crosswords, knitting, listening to the radio, looking at photographs, reading newspapers, playing solitaire and watching TV.” (Nygaard). Passing the time with simple pleasures such as having “’cups of tea’, ‘cigarette smoking’, ‘a sunny day’, ‘the odd chat’ or ‘someone giving me an ice-cream’”, according to Cahill, “brought much gratification”. This indicates some staff (or possibly visiting family) understood the residents well enough to facilitate these things when the residents may no longer be in the position to do without assistance like provide a pack of cards or to help position them near a window.

In contrast, the evidence also demonstrated that some residents found their lives mundane and the organised activities boring.

‘‘What’s making me drum? The lack of life.’’ (CG03) Clare

“Nothing. That’s what it is. When you’re just there, it’s boring in the long run. Very little happens.” (P29) Nygaard

Some of the feelings of boredom can be explained by people not having enough to do, and a lack of facilitation of preferred or meaningful activities by staff or family.

“But nobody knows that I want a job.” (GW02) Clare

Not being satisfied with the activities on offer and the lack of choice was another reason to feel bored.

“I listen to that music, but to be perfectly honest, I am not so fond of this music being played. ... I like to hear the accordion, I do.” P8 Nygaard

“Do you like the activities that (NH) organizes for you?” “No I don’t because I didn’t pick these out.” (female, 83) Cahill

Reduced freedom or opportunity to get out was a source of frustration.

“(Staff) won’t let me out as I am not fit to walk.” (female, 85) Cahill

“I must not go alone. I’ve gotten that printed I don’t know how many times”. (P13) Nygaard.

A lack of awareness or options for activities could also have influenced the perception that there was nothing to do.

“I’ve never heard about it.” (female, 92) Cahill

##### Environment

Residents felt comfortable and content when they had private space away from other residents, where they could rest, feel secure and quiet, and be themselves.

“I’ve got my own room. I didn’t think it was true. I feel like a countess!” (P16) Nygaard

“And I’m perfectly safe, the door is not locked, and I’m not afraid that someone may come who doesn’t belong here”. (N14) Nygaard

They knew the current setting was not their past home but spoke about having possessions in their rooms to make them feel homely, and to help remind them of the people that were close to them, and to a degree, remind them of who they were.

“A small little house can be just the size that is nice to have for the things you have. I also hung up some pictures to remember those in the family.” (P14) Nygaard

Finding a way to link to themselves and to their past by having personal belongings around them could be a source of comfort, and a way of gaining some control over their environment and the losses they experienced.

#### How People Feel About Themselves

##### It’s Not Me

A recognition of the changes in their living situation and themselves over time affected self-esteem and self-worth. Noticing changes in themselves could lead to people with dementia feeling sad about their losses, wishing they could do more, and having a weaker sense of their own identity. This was evidenced in self-deprecating language. Not feeling as capable as before could be associated with feelings of failure.

‘‘I’ve failed an awful lot now here.’’ (BG03). Clare

“I don’t know, but it is not me anyway. I like to do so much, and suddenly I am an old hag…I am so old (crying) that I couldn’t do anything anymore.” (Mina) Mjørud

A weaker sense of identity was linked to changes in how they spent their time, and the reduced opportunities for activities in the care home.

“I’d rather be doing something, yes, al . . . although I . . . I . . . I . . .there’s not a lot I can do, I’m capable of doing.’’ (AL03) Clare

“…and it is difficult because it is much the same that recurs”. (Betty) Mjørud

One paper reported distressing thoughts linked to reflecting about the future.

“I have nothing to look forward to only carry on till my life ends.” (female, 87) Cahill

##### I Still Can

In contrast to feeling “it’s not me”, there were examples of people expressing optimism and satisfaction with themselves and what they could do, even though they also recognised that on balance, they had experienced losses. This was achieved by reflecting positively on the past, their achievements, and their current abilities.

“I can still do everything I make me mind to...I read all, well, when I can get hold of a book, I read all the time, dear, I’ve always read ever since I was old enough to learn to read.” (GW01) Clare

Thinking they could continue to do things and ‘not giving in’ (Nygaard, Clare) could be interpreted as providing a sense of control over their dementia, and some hope for the future. How these positive expressions correspond to what people actually do and how they feel on a day-to-day basis is unknown, but they may be a method of coping and remaining hopeful.

“I’m th … thankful for what I can do, you know what I mean, I won’t give in.’’ (FP01). Clare

Feeling positive about themselves could be assisted by noticing their situation was not as bad as others. Downward comparisons to other people in the care home could contribute to feelings of gratefulness.

‘‘I can do with me own company, which is something, it must be very awful if they come to be on their own and there’s nobody there but themselves and they, it must be terrible must that… It must be very sad for people who haven’t any family.’’ (BG06) Clare

“Not everyone can [talk] even, and I feel sorry for them as it must be awful to be unable to talk to people as that is how you express yourself.” (P10) Nygaard

Making downward comparisons could help people with dementia realise that things could be worse, but of course, seeing others who had more advanced symptoms of dementia could be distressing, as it could make them think that this was their fate. As mentioned previously in the section ‘How people feel about the care home itself’, people with dementia were sometimes physically or emotionally distanced from other residents. This could be a coping strategy to avoid being around other people with more advanced dementia because it is distressing or irritating or could simply reflect feeling distanced from people in general.

## Discussion

This is the first systematic review and qualitative synthesis of studies reporting the subjective experiences and views of well-being and quality of life of people with moderate-to-severe dementia. There were only a few studies that included direct quotes from people with moderate-to-severe dementia, and no studies were identified that were published after 2020. Our evidence demonstrates a variety of experiences and responses to the environment, differences in well-being and coping strategies, and indicates the complexity of individual circumstances. We observed that people could rationalise, make comparisons, and identify ways to manage their well-being. They found ways to feel better about themselves by comparing their past to their current lives and comparing themselves with other people. They were impacted by the people around them and sought privacy as well as activities with others to reduce boredom. Activities, social interactions and familiar objects could help to remind them of their connections to others, their past lives and themselves, and could be sources of enjoyment or pleasure.

The identified themes have a common thread. They demonstrate a need for people with moderate-to-severe dementia to maintain a positive sense of self. A positive sense of self for people living in care homes has previously been found to be a fundamental necessity for good quality care, facilitated by meaningful engagement and opportunities to feel useful and valued (Milte et al., 2016). Other studies have found that amongst people with moderate-to-severe dementia, loss of autobiographical memory serves to compromise well-being primarily because of loss of identity. However, this is not a clear linear relationship as positive associations with life-satisfaction amongst those with severe dementia have been observed (Jetten et al. 2010). Indeed, others have found that personal identity can remain intact in people with severe dementia (Batra et al. 2016, Cohen-Mansfield et al., 2000; Kitwood, 1993; Sabat & Harré, 1992). In our study, a need for a sense of self was sought through maintaining connections to people, which could help to support a social persona (Sabat & Harré, 1992) or their autobiographical identity (Kłosińska & Leszko, 2023). In the studies identified, people with dementia maintained social connections to the people closest to them either face-to-face, by having photos to remind them, or by reminiscing.

Spending time with family or friends was an opportunity to lift mood and allowed a connection to themselves as members of a family. This, and the finding of the importance of attachment to personal belongings, can be interpreted as ‘productive nostalgia’, where evoking sentimental feelings from items and the people and memories associated with them can enhance positive feelings about oneself and a sense of connectedness (Cheston et al., 2015). People with dementia have previously reported the impact of the care home feeling ‘homely’ (an example of productive nostalgia) on their well-being (Rijnaard et al., 2016, Sheills et al., 2020; O’Rourke et al., 2015). Our findings demonstrate this remains true for people with more advanced dementia.

The studies showed how people with more advanced dementia reflected on what it meant to move into residential care. Comparing the care home to their previous home, reduced contact with family, and experiences of loss in choice and autonomy indicated negative impacts of the changes in their social situation. IDEAL findings showed the importance of social situation and social resources in perceptions of living well for people with mild-to-moderate dementia (Clare et al., 2019). For our population, changes in social situation were a cause of dissatisfaction, but we also discovered the ability to self-reflect and think positively despite experiencing losses, and the application of coping skills to adapt to their situation to maintain quality of life. This supports the IDEAL model of living well (Clare et al., 2019). The model proposes that ‘living well’ is manifest in the balance between resources, in the form of previous experiences and abilities and assets, and the experience of the personal, physical, social, and practical challenges being faced. For example, wrestling with their loss of home and community and rationalising their current situation could be seen as a way of coping with reduced social networks, reduced access to social activities, and resulting change in social status. Living in a care home could be accepted if they believed they needed help and assistance, and felt safe and cared for. Feelings of abandonment and loneliness could be managed by focusing on emotional attachments to important people in their lives.

All studies reported experiences of boredom and the importance of meaningful engagement. This review highlights the connection between boredom with feeling worse about themselves. Other studies have demonstrated degrees of boredom in a single care home, indicating the personal nature of boredom (Gebhard & Frank, 2024). It must be noted that activities did not need to be participatory or organised. There were also expressions of well-being when time was spent alone or relaxing, indicating the importance of choice in life, which supports the findings of O’Rourke et al. (2015). To offer choice requires staff to be attentive to individual preferences and dislikes, to find activities that encourage feelings of capability, purpose, usefulness or helpfulness, and for staff to have flexibility to be creative in adapting activities when the person with dementia lacks personal agency to initiate activities. To create moments of pleasure or productive nostalgia would require insight and creativity by staff, for example, through music, smells, possessions, or objects. Unfortunately, amongst the positive accounts of staff being ‘nice’ and ‘caring,’ and examples of people with dementia engaging in their preferred pleasurable and meaningful activities, we also found evidence that people with dementia lacked stimulation, were often bored and felt worthless. Kitwood (1993) argued that people with dementia may retain the aspects of themselves associated with personal identity but may lose those aspects that require validation by others. Sabat and Harré (1992) have noted that “the primary cause of the loss of self in persons with Alzheimer’s dementia is the way in which others view and treat them.” (Sabat & Harré, 1992, p. 453) All studies provided recommendations for improvements in care in the form of meaningful options for engagement, and better relationships and understanding of residents.

Positive and negative self-reflection were evident in how people spoke about themselves. This manifested in whether they believed they felt capable and if they had a positive attitude that helped them make the most of their situation. We have previously showed that the ability to manage everyday life influenced psychological health in people with mild-to-moderate dementia (Clare et al., 2019). It is important to note that views of ‘I still can’ may not have been based on reality for some, and awareness of one’s abilities and situation is known to vary in people with advanced dementia O’Shaughnessy et al. (2021). However, remaining optimistic and ‘creating’ a positive view of personal capability, purpose, usefulness, or helpfulness may be a useful psychological coping strategy that helps to maintain well-being. Therefore, assisting the person to maintain a positive view of themselves, whether based on their experience or not, is likely to be worthwhile for the person with dementia to help maintain a positive identity.

### Limitations

The participants lived in long-term residential care settings, so we do not know about the subjective views and experiences of quality of life for people living in their own homes. None of the studies identified participants as LGBTQ+ individuals or belonging to any minority ethnic groups. Although any experiences of inequality or discrimination in people with moderate-to-severe dementia are likely to be salient influences on well-being, this possibility could not be explored.

There were several methods of assessing dementia severity within the included studies, and potential differences in the classification of severity. Also, one paper included people with mild dementia, so it was essential to focus the analysis on the direct quotes from people who were known to have moderate-to-severe dementia. Even though we attempted to analyse these independently of the findings provided by authors, it was impossible not to be influenced by the included paper authors’ interpretations of their data.

The included studies focused on the experience of people with dementia across 20 residential care homes. However, due to limitations in the reporting of contextual information, we were unable to comment on factors that may have shaped residents’ subjective perceptions of well-being and quality of life—such as the organisational structures of the care settings or the professional backgrounds and qualifications of care staff.

### Implications

Interest has been abundant in understanding the views of people in care homes to identify unmet needs (Schweighart et al., 2022, Sheills et al., 2020) with a view of improving quality of care, but fewer attempts to research of the subjective views of quality of life held by people with more advanced dementia. Understanding the unique perspectives of people with more advanced dementia is the starting point to prioritisation of emotional as well as practical care and support a person needs, but it may be perceived as challenging in practice. It is acknowledged that communication with people with more advanced dementia requires compassion, patience, and good communication skills (Collins et al., 2022). This review demonstrates the usefulness of adopting typical qualitative methods to elicit the views of people with moderate-to-severe dementia, namely semi-structured interviews and open-ended questions in exploratory conversations, however, additional communication support may be needed as dementia progresses further, for example, using picture-based communication tools (Collins et al., 2024).

Evidence presented in this paper demonstrates that there are modifiable influences on quality of life of people with moderate-to-severe dementia. Finding ways to help people with moderate-to-severe dementia have choices in how they spend their time, who they interact with, and their environment is likely to be beneficial.

Depending on the existing skills and experience of residential care staff, and the kinds of strategies they are using, it may be possible that staff could benefit from communication training to assist them in facilitating positive interactions with people with dementia. It is also possible that allowing staff more time and opportunities to interact with residents and increasing opportunities for positive interactions with visitors and the wider community, whether in person, virtually or through positive reminiscence may help people with dementia to experience positive well-being. Interventions to reduce boredom need to be personalised, and identification and management of easily modifiable issues that influence engagement in activities such as managing pain (Harding et al., 2024) could be of value.

## Conclusions

Despite a shift in language and policy over the last 30 years, little progress has been made to understand influences on quality of life amongst people with moderate-to-severe dementia from their subjective perspective. To further our current knowledge of this area, research is needed to ascertain the degree to which, and ways in which, residential care homes are attempting to understand well-being amongst care home residents with more advanced dementia, and how positive well-being is facilitated. It would be helpful to identify the most appropriate methods used to measure and monitor the subjective well-being of people with moderate-to-severe dementia and useful strategies for staff to minimise boredom and maintain a positive sense of self.

It must be more widely understood that it is (a) possible and (b) important to gain the subjective views of people with moderate-to-severe dementia and (c) quality of life can be and is worth preserving in this group. We call for a humane shift towards prioritising quality of life for each resident using a personalised approach to their well-being.

## Supplemental Material

Supplemental Material - Factors Affecting Well-Being and Quality of Life From the Perspective of People With Moderate-To-Severe Dementia: A Qualitative Evidence Synthesis Supplemental Material for Factors Affecting Well-Being and Quality of Life From the Perspective of People With Moderate-To-Severe Dementia: A Qualitative Evidence Synthesis by Claire Pentecost, Anna Carr, Catherine M. Alexander, Linda Clare in Dementia

## Data Availability

The data that support the findings of this study are available from the corresponding author upon reasonable request.*
