# Supplementary material for: Factors Affecting Well-Being and Quality of Life From the Perspective of People With Moderate-To-Severe Dementia: A Qualitative Evidence Synthesis
Source: Dementia (London). 2025 Dec 29;25(8):2037–58. doi: 10.1177/14713012251410993 (PMC13683893; doi:10.1177/14713012251410993)
Supplement: Supplementary file 1 [file sj-pdf-1-dem-10.1177_14713012251410993.pdf]

Supplemental file 1: ENTREQ checklist

**Item**	**Guide and description**	**Reported on page #**
Aim	State the research question the synthesis addresses	3
Synthesis methodology	Identify the synthesis methodology or theoretical framework which underpins the synthesis, and describe the rationale for choice of methodology (e.g. meta-ethnography, thematic synthesis, critical interpretive synthesis, grounded theory synthesis, realist synthesis, meta-aggregation, meta-study, framework synthesis).	4
Approach to searching	Describe the information sources used (e.g. electronic databases (MEDLINE, EMBASE, CINAHL, psychINFO, Econlit), grey literature databases (digital thesis, policy reports), relevant organisational websites, experts, information specialists, generic web searches (Google Scholar), hand searching, reference lists) and when the searches were conducted; provide the rationale for using the data sources.	4
Inclusion criteria	Specify the inclusion/exclusion criteria (e.g. in terms of population, language, year limits, type of publication, study type).	4
Data sources	Describe the information sources used (e.g. electronic databases (MEDLINE, EMBASE, CINAHL, psychINFO, Econlit), grey literature databases (digital thesis, policy reports), relevant organisational websites, experts, information specialists, generic web searches (Google Scholar), hand searching, reference lists) and when the searches were conducted; provide the rationale for using the data sources.	Supplemental file 2
Electronic Search strategy	Describe the literature search (e.g. provide electronic search strategies with population terms, clinical or health topic terms, experiential or social phenomena related terms, filters for qualitative research and search limits).	Supplemental file 2
Study screening methods	Describe the process of study screening and sifting (e.g. title, abstract and full text review, number of independent reviewers who screened studies)	4-5
Study characteristics	Present the characteristics of the included studies (e.g. year of publication, country, population, number of participants, data collection, methodology, analysis, research questions).	Supplemental file 3
Study selection results	Identify the number of studies screened and provide reasons for study exclusion (e.g. for comprehensive searching, provide numbers of studies screened and reasons for exclusion indicated in a figure/flowchart; for	P 6-7 & figure 1

	iterative searching describe reasons for study exclusion and inclusion based on modifications to the research question and/or contribution to theory development).	

Rationale for appraisal	Describe the rationale and approach used to appraise the included studies or selected findings (e.g. assessment of conduct (validity and robustness), assessment of reporting (transparency), assessment of content and utility of the findings).	5
Appraisal items	State the tools, frameworks and criteria used to appraise the studies or selected findings (e.g. Existing tools: CASP, QARI, COREQ, Mays and Pope [25]; reviewer developed tools; describe the domains assessed: research team, study design, data analysis and interpretations, reporting).	5,7
Appraisal process	Indicate whether the appraisal was conducted independently by more than one reviewer and if consensus was required.	5
Appraisal results	Present results of the quality assessment and indicate which articles, if any, were weighted/excluded based on the assessment and give the rationale.	7, table 1
Data extraction	Indicate which sections of the primary studies were analysed and how were the data extracted from the primary studies? (e.g. all text under the headings "results /conclusions" were extracted electronically and entered into a computer software).	5
Software	State the computer software used, if any.	5
Number of reviewers	Identify who was involved in coding and analysis.	6
Coding	Describe the process for coding of data (e.g. line by line coding to search for concepts).	6
Study comparison	Describe how were comparisons made within and across studies (e.g. subsequent studies were coded into pre-existing concepts, and new concepts were created when deemed necessary).	6
Derivation of themes	Explain whether the process of deriving the themes or constructs was inductive or deductive.	6
Quotations	Provide quotations from the primary studies to illustrate themes/constructs, and identify whether the quotations were participant quotations or the author's interpretation	Table 3, p 14-20
Synthesis output	Present rich, compelling and useful results that go beyond a summary of the primary studies (e.g. new interpretation, models of evidence, conceptual models, analytical framework, development of a new theory or construct).	Table 3, p 14-20

Supplemental file 2 Search terms

	Dementia type		Severity and setting		Quality of life or well-being		Qualitative*
Search terms	(Alzheimer* or dement* or lewy or fronto* or confus* or sundown* or "cognitiv* impair*") ti,ab,kf	AND	(severe or moderate or advanced or "nursing home*" or "long term care" or "long-term care" or "assisted living" or "residential" or "care home*" or respite or "day care" or "memory care" or institutionali*) ti,ab,kf	AND	("quality of life" or qol or "life quality" or wellbeing or "well being" or well-being or "life satisfaction" or "satisfaction with life" or "personal satisfaction" or hrqol or happiness or happy or joy or enjoy* or "experience*" or dignity or (meaning adj3 life) ti,ab,kf	AND	(focus groups/ or interviews as topic/ or narration/ or qualitative research/ or ((face or f2f or guided or depth or indepth or informal or semistructured or structured or unstructured) adj4 (discussion* or interview* or questionnaire*)).ti,ab,kf. or (ethnograph* or (field adj1 work) or fieldwork or (focus adj1 (group or groups)) or (key adj1 (informant or informants)) or qualitative) ti,ab,kf

Searched on 21st March 2025 in OVID with the following databases selected: Embase, Ovid MEDLINE, APA PsycInfo, Social Policy & Practice
*Qualitative filter terms were derived from advice found on the InterTASC Information Specialists' Sub-Group Search Filter Resource for qualitative research https://sites.google.com/a/york.ac.uk/issg-search-filters-resource/home/qualitative-research.

Supplemental file 3: Descriptive data

lead author, country, year	Aim	Methodological orientation or theory/data collection method/analytical method	Framework for questions	Setting and context	Details of who collected data	Moderate to severe dementia included in qualitative analysis/total recrited	Mean age (total) mean age (moderate to severe subgroup)	Number of males	*Assessment of dementia severity	Reported dementia severity	Reported themes (whole study)
Cahill, Ireland, 2011	To examine if differences or similarities exist in perceptions of quality of life amongst nursing home residents with different levels of cognitive impairment.	none given/interviews/thematic analysis	sources of happiness and sadness, privacy, homeliness, activities, interactions with fellow residents and staff, what residents favoured most about living in long-term care and what they liked least	three nursing homes	researcher (no further details)	48/61	85.3 / 86.3	13	MMSE	13 mild, 20 moderate, 28 severe MMSE 0-10 mean 5.75	Main study themes were 1) social contact (including sub-themes of family, residents and staff 2) attachment (including sub-themes of feeling at home/missing home and connectedness) 3) pleasurable activities (including sub-themes of structured vs unstructured activites, outings and momentary pleasures) 4) affect (including the sub-themes of happiness, sadness/depression and lonliness). The narratives of those with a severe cognitive impairment reflected an absence of social contact, a quest for human contact and a lack of awareness of structured pleasurable activities. A large majority also reported feelings of loneliness, isolation and a search for home.
Clare, Wales, 2008	To explore the subjective experience of life with dementia in residential care in a large sample of residents, to understand the psychological impact of being in this situation, and to develop a thematic framework for making sense of this experience that could contribute to improving quality of life and quality of care.	phenomenology and symbolic interactionism (Smith Jarman and Osbourne 1997)/unstructured conversations/interpretative phenomenological analysis	conversations were unstructured and led by the participants themselves	ten residential care homes in England and Wales specialising in the care of people with dementia, for older people with designated sections devoted to the care of people with dementia, or homes with a mixed resident group	researcher (no further details)	71/71	83.4	12	MMSE	MMSE from 0 to 20 (M=9.76, SD=6.66). Over half of the respondents scored 11 or fewer points.	Four themes, labeled in the participants' own words (with prevalence rates among the 80 participants given in parentheses), were as follows: ''Nothing's right now'' (89% of participants); ''I'm alright; I'll manage'' (76% of participants); ''I still am somebody'' (76% of participants); and ''It drives me mad'' (48% of participants).
Mjorud, Norway, 2017	To investigate the personal experience of living in a NH over time and what makes life better or worse from the perspective of the person with dementia.	phenomenological hermeneutic study/interviews/phenomenological hermeneutic analysis	questions were based in prior research literature on quality of life and theory on person centred care: Thriving Occupational or meaningful activities Relationships. Questions about whether they felt content, whether they thrived and whether they felt safe. The rest of the questions were all follow-up questions based on the answers from the informants	three nursing homes	a registered nurse with clinical experience from nursing homes	11/12	84.9	10	CDR	n=12. 10 scored 2 CDR, 1 scored 3 CDR, 1 scored 1.	Four themes: "Being in the nursing home is okay, but you must take things as they are"; "Everything is gone"; "Things that make it better and things that make it worse"; "Persons – for better or worse? Staff, family, and co-residents".
Nygaard, Norway, 2020	To explore what matters to nursing home residents with dementia based on their perceptions of nursing homes as home.	none given/unstructured interviews/thematic analysis	conceptual framework: 'nursing homes as home.' The researcher had a general topic in mind but formatted many specific questions as the interviews proceeded in response to what the residents said to encourage them to answer at length and in vivid detail	three nursing homes in a large municipality in Norway. Interviews collected October 2018-April 2019	a nurse and PhD student with previous experience in dementia care	35 no severity mentioned but assume all based on description of residential care setting	86.2	10	none	no	One over-arching theme "tension between the experiences of a nursing home being a home and an institution" and five themes; "myself and my relationships with fellow residents", "creation of individualised living spaces", "single rooms with personal decor that enhances a sense of connectedness", "transition between the old home and the new home" and "significant activities providing meaning".
Verloo, Switzerland, 2018	To examine the quality of life among cognitively impaired older adults (CIOA) living in nursing homes.	deductive content analysis/interviews/deductive content analysis	questions matched to the specific domains of the QoL-AD-fv hierarchical framework for Quality of Life in dementia described by Jonker et al. using open-ended questions concerning environmental and personal factors e.g. "What do you think influences your quality of life?"	one nursing home. The study population was composed of CIOAs living in the psychiatric unit of one nursing home in French-speaking Switzerland, All CIOAs (and their legal representatives), men and women who had been living in the unit for at least two weeks, were invited to participate in the study. CIOAs in end-of-life situations were excluded. The study nurse recruited participants in collaboration with the psychiatric unit's nursing supervisor. Recruitment took place between January and September 2016.	no data	15/15	86	5	MMSE	all CDR=3	Four themes 1) human dignity and acceptance. 2)development and existence. 3) Functionality and health. 4) Recognition and safety.

*Mini-Mental State Examination (MMSE) (Arevalo-Rodriguez et al. 2015): scores 0-30. 10-18=moderate dementia, ≤9=severe impairment. Clinical Dementia Rating (CDR) scoring: 2=moderate 3=severe dementia

Supplemental file 4 Quality assessment details

Lead author and year	1. Was there a clear statement of the aims of the research	comment	2. Is a qualitative methodology appropriate	comment	3. Was the research design appropriate to address the aims of the research	comment	4. Was the recruitment strategy appropriate for the aims of the research	comment	5. Was the data collected in a way that addressed the research issue	comment	6. Has the relationship between the researcher and the participants been adequately considered	comment	7. Have ethical issues been taken into consideration?	comment	8. Was the data analysis sufficiently rigorous	comment	9. Is there a clear statement of findings?	comment	10. How valuable is the research?	comment	Number of yes, unsure, no	Agreed score from a possible 10
**Cahill, 2011**	yes	goal is important and relevant, wanted to examine if differences or similarities exist in perceptions of Quality of Life amongst nursing home residents with different levels of cognitive impairment	yes	uses a qualitative approach to elicit peoples' own subjective views and individual accounts about their Quality of Life	yes	to elicit subjective views	yes	care homes where there people with different dementia severity can be found	yes	semi-structured interview schedule questions provided	yes	no mention of the interaction between the participant and the researcher, but ethical issues have been considered	yes	approval and consent described	yes	description of coding and emergence of themes and examples provided	yes	description of themes and sub themes, with explanation	yes	included people with severe dementia and used interviews, good insights and illustrative quotes	yes=9 unsure=1	9
**Clare, 2008**	yes	goal and relevance is clear. Elicited views directly from the residents themselves to understand the psychological impact of living with dementia in long-term care	yes	also uses quantitative data to demonstrate numbers of people with different Mini-Mental State Examination scores and how this may have had relevance to the types of themes	yes	discussion of rational for choosing Interpretative Phenomenological analysis	yes	selected from care homes	yes	saturation not mentioned but large sample	unsure	the article says they have done this but not how	unsure	ethical approval for the analysis of this data is given, but no mention of the ethical issues for the study in which the conversations were collected	yes	good description and clear how themes were derived, and good amount of quotes	yes	good section on credibility	yes	contribution to new knowledge, implications and transference to other population discussed	yes=8 , unsure=2	8
**Mjorud, 2017**	yes	no previous Norwegian studies. No studies combined interviews with observations. Few used follow-up conversations.	yes	plans to investigate personal experiences	yes	unstructured interviews, with initial prompt questions, then follow on questions led by participant responses	yes	People with dementia who had lived in a nursing home for a maximum of six months and with a life expectancy of more than three months	yes	good description of questions asked and prompting, setting, data collection, methods well described	no	registered nurse researcher with clinical experience from nursing homes met with each informant twice approximately three months apart, but bias not explicitly mentioned	yes	approval and issues highlighted and addressed	unsure	no explanation of how themes were identified.	yes	explicit, discussion of evidence, more than one researcher	yes	good consideration of gaps and contribution	yes=8 , unsure=2	8
**Nygaard, 2020**	yes	describes the conceptual framework : sense of home can be disrupted by frailty, and the importance and implications for practice and policy.	yes	unstructured interview guide and interviews conducted in the home is appropriate to illuminate the subjective experiences of participants, and qual is the correct methodology	yes	yes exploratory qualitative design is appropriate. No more information.	yes	not explained in full what criteria was used to identify potential participants but appropriate they were recruited from nursing homes and they were long term residents, may have been bias in selection, but this is not clear	yes	setting, how data was collected - extended conversations, methods were explicit and saturation is explained	unsure	interviewer is a nurse and student and previous experience n dementia care but their role and potential bias is not mentioned, but useful to know the background in dementia care re had skills to 'formatted many specific questions as the interviews proceeded in response to what the residents said to encourage them to answer at length and in detail'	yes	large paragraph on ethical approval, including funding, informed consent, assent, and ongoing consent	yes	stages of analysis clearly described, more than one analyst, suitably qualified (credibility), discussion, independent coding and agreement, clear theme development, sufficient data to support the findings in tables. no reflexivity, but process was clear and agreement between analysists and co-authors.	yes	confusion if there are 4 or 5 themes, seems to be an issue with a table otherwise themes are well described in the findings but short summary is missing. clear and evidence to substantiate is provided with several quotes and discussion. Credibility discussed in methods section	yes	no reflexivity but otherwise good, and limitations are pointed out.	yes=9 unsure=1	10
**Verloo, 2018**	yes	qualitative component aim described : Explore Quality of Life among people with dementia living in care homes. Including objective, functional and subjective components in keeping with World Heath Organisation definition	yes	For phase II, the qualitative part of study.	yes	deductive content analysis, to increase understanding of issues around Quality of Life that had also been assessed objectively, and integrate the results	yes	men and women who had been living in the psychiatric unit of one nursing home for at least 2 weeks	yes	semi-structured interviews. Interviews recorded.	unsure	No indication of this.	yes	approval consent and confidentiality described	unsure	analysis steps are described but not clear how themes were derived from the data, some presentation of the data (quotes) with findings, no comment on the researchers role	yes	no credibility of findings mentioned i.e. other researchers, but 'discussion' is mentioned, and more than one researcher collected data, yes findings discussed in relation to the research question	yes	yes good contribution to knowledge, and implications, state caution of transferability.	yes=8 , unsure=2	8
